# Enhancing doping contrast and optimising quantification in the scanning electron microscope by surface treatment and Fermi level pinning

**DOI:** 10.1038/s41598-018-22909-2

**Published:** 2018-03-27

**Authors:** Augustus K. W. Chee

**Affiliations:** 1 Centre for Advanced Photonics and Electronics, Electrical Engineering Division, Department of Engineering, University of Cambridge, 9 JJ Thomson Avenue, Cambridge, CB3 0FA United Kingdom; 2 Department of Materials Science and Metallurgy, University of Cambridge, Pembroke Street, Cambridge CB2 3QZ, United Kingdom

**Keywords:** Characterization and analytical techniques, Scanning electron microscopy, Imaging techniques

## Abstract

Recent advances in two-dimensional dopant profiling in the scanning electron microscope have enabled a high throughput, non-contact process diagnostics and failure analysis solution for integrated device manufacturing. The routine (electro)chemical etch processes to obtain contamination-free, hydrogen-terminated silicon surfaces is industrially important in ULSI microfabrication, though doping contrast, which is the basis for quantitative dopant profiling, will be strongly altered. We show herein that ammonium-fluoride treatment not only enabled doping contrast to be differentiated mainly by surface band-bending, but it enhanced the quality of linear quantitative calibration through simple univariate analysis for SE energies as low as 1 eV. Energy-filtering measurements reveal that the linear analytical model broached in the literature (*c.f*. Kazemian *et al*., 2006 and Kazemian *et al*., 2007) is likely to be inadequate to determine the surface potential across semiconductor *p-n* junctions without suitable deconvolution methods. Nevertheless, quantification trends suggest that energy-filtering may not be crucial if patch fields and contamination are absolutely suppressed by the appropriate edge termination and passivation.

## Introduction

Secondary electron (SE) doping contrast has hitherto been widely adopted as a scanning electron microscope (SEM) metrology standard in several phases of semiconductor manufacturing; this technique is instrumental in, *inter alia*, physical and failure analysis^1–3^, critical dimension mapping and control^4^, and process optimisation^5^ to implement new generations of intensely reliable and energy-efficient integrated circuits and devices. Upon irradiating a specimen with an energetic stream of primary electrons, ions or photons, the resulting low energy electron emissions via inelastic scattering when the ionisation potential is exceeded constitute the SE signal. At a *p*-*n* junction, the SE signal is strong from the *p*-region and weak from the *n*-region under standard SEM imaging conditions. *P*-regions appear bright and *n*-regions dark in semiconductor circuits, producing the so-called doping contrast, which is versatile as a voltage contrast technique of considerable scientific and technological importance. This sharp brightness transition can be used, sometimes in conjunction with EBIC analysis^6^, to evaluate electrical properties of doped homo- or hetero-junctions. Increased sensitivity can be achieved by external reverse biasing even under high magnification^2,4,7–10^, albeit trading off resolution^10^. Demonstrations of SE imaging capability have also recently reached an unprecedented level with visualisation of the crystal lattice and individual atoms in the bulk and surface^11^, rivalling that in the scanning tunnelling electron microscope^12^. Owing to an instrumental ability to generate sub-nanometre ultrafine probes and obtain highly-localised secondary emissions, doping contrast at atomic-scale resolution may be possible, especially for heavy atoms^11^.

Although an initial drawback of the SEM technique is the dearth of well-calibrated quantitative models, progress has been made in evaluating and modelling the doping contrast mechanism in bulk materials, which is due to the built-in potential across the electrical junction, modified by the effects of surface band-bending, surface boundary scattering, the local electric fields above the specimen surface called patch fields, and the detector collection solid angle^13^. Where the crystal is terminated at the surface, the abrupt potential change results in the formation of a surface dipole layer, which for a semiconductor, extends below the surface giving rise to a surface depletion region and surface band-bending^14–16^. The charges on a clean and ideal surface give rise to states, typically in the band gap, called Shockley or Tamm states; and surface states also arise from an oxide overlayer. Moreover, any variation in the two-dimensional electric potential landscape due to a non-uniform surface charge distribution can result in patch fields outside a specimen^17,18^. SEs generated in semiconductor crystals that diffuse to the surface therefore either encounter a diffusion barrier or drift through sub-surface electric fields due to the surface depletion region and dipole layer before percolating through surface/interface boundaries that modify the trajectories of emission^13,19^, and may be further subjected to refraction by any patch fields after escaping the surface^13,20^. The SE signal intensifies if the doping-dependent field and scattering effects enhance the collection efficiency into the lens bore, and vice versa.

Optimisation and quantification schemes are thus continually being developed and deployed to exploit the SE characteristics for rapid, contactless and non-destructive dopant profiling in the SEM at high spatial resolution. Notably, energy-filtered imaging allows improved quantitative features, reproducibility, sensitivity and accuracy^9,21–26^. Further, by acquiring an image series through an energy window of interest, *in situ* SE spectromicroscopy mandates an additional dimension permitting evaluation of the surface electronic band structure via kinetic energy (*E*_SE_) constituents of the composite signal^22,27^. However, it is known that the inherent and heretofore unresolved limitations in the interpretation of SE signal measurements represent a major source of quantification error. For instance, adlayer-semiconductor contacts formed from adventitious hydrocarbons alter the surface dipole layer and contribute to doping contrast^28^ but lead to stochastic uncertainty in the final analysis^24,27,29^. Hence, quantitative dopant profiling cannot be reliably performed with sufficient sensitivity and accuracy unless the near-surface effects, including that of surface band-bending and patch fields, are well controlled and well correlated with changes in doping contrast. Analogous to other surface-sensitive characterisation techniques such as absorption spectroscopy or X-ray photoemission spectroscopy^30^, doping contrast will be strongly modified by (electro)chemical processing methods that are routinely employed for ULSI microfabrication, or have potential utility in single dopant electronic device fabrication^31^. Therefore in this study, we examine and demonstrate in the SEM for the first time, energy-filtered SE intensity and spectromicroscopy measurements from a wide semiconductor doping range, not only before, but after wet chemical etching. Herein, we report on doping contrast characterisation from silicon specimens that were treated reproducibly using CMOS electronic grade ammonium fluoride (NH_4_F) solution.

The NH_4_F-treatment at room temperature is known to etch the surface, removing the native oxide layer and any organic contamination, and produce clean and atomically flat or vicinal silicon surfaces having ideal hydrogen atom surface termination and passivation that is stable in air, and known to last several hours or up to hundreds of days^32,33,34^. Single-domain silicon monohydride passivation can be achieved, as opposed to the generation of co-existing monohydride-, dihydride- and trihydride-terminations that underpin a microscopically rough surface topology if using aqueous hydrofluoric (HF) solution^32,35^. Recent results^27^ have established that the consequential doping contrast change follows an evolution of charge states and an increased surface state density and surface band-bending which suppresses patch fields and isolates surface band-bending effects on doping contrast. We will show using a combination of specialised energy-filtering and *in situ* SE spectromicroscopy techniques, that by avoiding or minimising possible sources of quantification errors *viz*. patch fields, residual contamination, surface roughness, *etc*., the effect of the surface-treatment on doping contrast is to promote quantitative dopant profiling over a wide doping and spectral range.

## Results

### Unfiltered doping contrast

**Figure 1:**
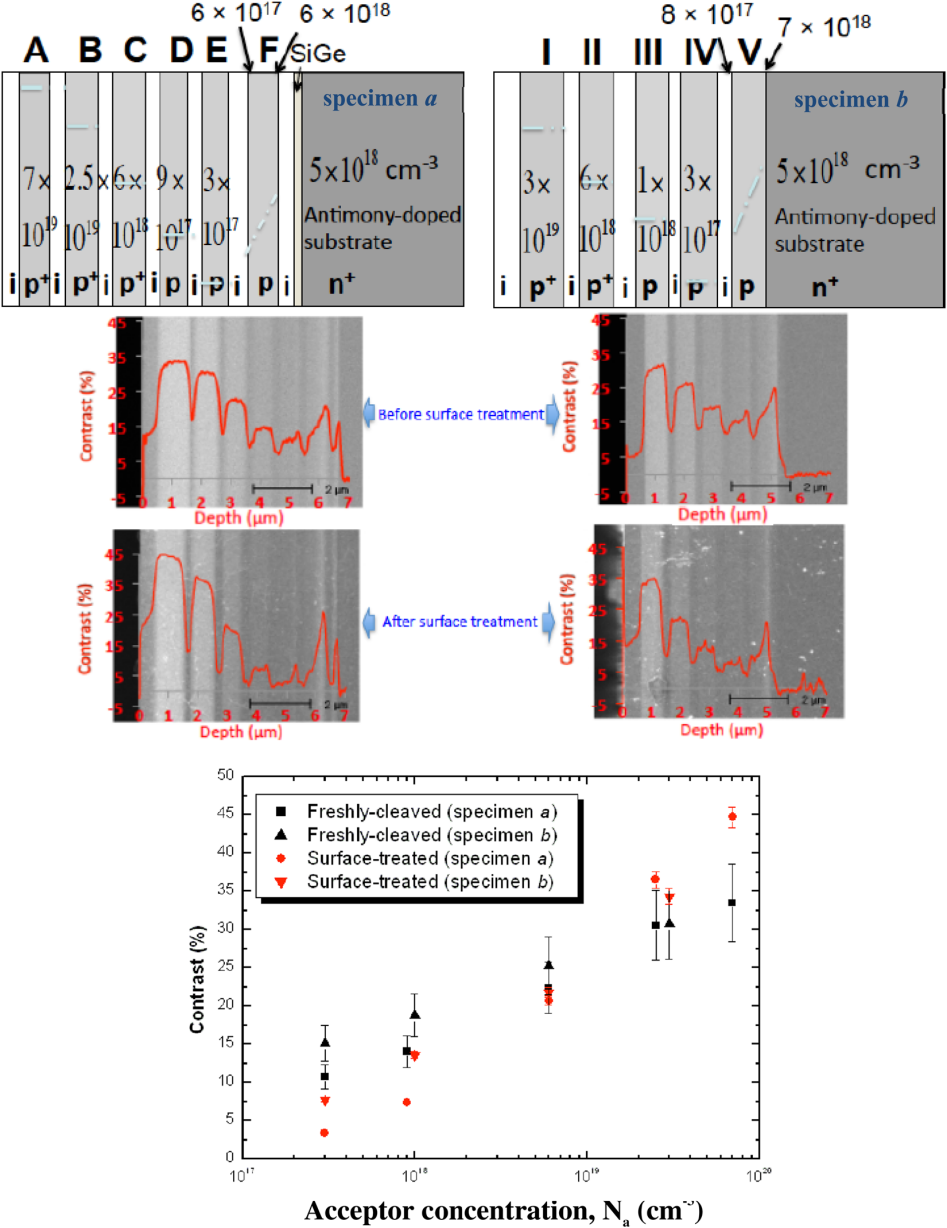
Doping contrast from silicon specimens before and after NH_4_F-treatment. (Top) Cross-sections of epitaxial structures comprising boron-doped silicon layers grown with (specimen a) or without (specimen b) a silicon-germanium marker layer on an *n*-type silicon substrate antimony-doped to 5 × 10^18^ cm^−3^. The dopant levels have been confirmed by SIMS measurements. (Middle) Unfiltered SE doping contrast images with depth profile overlays from freshly-cleaved and treated specimens. The white specks that appear after NH_4_F-treatment represent residual ammonium salts on the surface. Appropriate brightness and contrast settings were applied for the respective images to demonstrate strong doping contrast. (Bottom) Column-averaged doping contrast values from the *p*-layers of interest as a function of acceptor concentration, before and after surface-treatment. The dopant concentration varies steeply from ~6 × 10^17^ to ~6 × 10^18^ cm^−3^ across layer F (specimen *a*), and from ~8 × 10^17^ to ~7 × 10^18^ cm^−3^ across layer V (specimen *b*), hence these layers are not included in the data points. The experimental error for each data point is ±15% of the contrast value for the freshly-cleaved specimens and ±3% after surface-treatment.

Figure 1 compares the unfiltered doping contrast from abrupt homojunctions of freshly-cleaved and NH_4_F-treated silicon specimens. The extraction potential was 20 V and the working distance was ~6 mm, providing standard micro-analytical conditions for strong doping contrast and high signal-to-noise quality^8,26,29^. A native oxide surface layer, *ca*. 5–10 Å thick, is expected to form rapidly on the freshly-cleaved sample^36^, but will be removed by treatment with 40% NH_4_F which passivates the surface so that the oxide layer does not reform rapidly. The specimen was immediately inserted into the SEM and the SE images were recorded within ~10 min. after surface-treatment, thus no oxide overlayer is expected under clean, high vacuum conditions, consistent with *e.g*. refs^37,38^.

Whereas the doping contrast sensitivity to low N_a_ is unchanged by energy-filtering^26^, it reduced considerably after surface-treatment (Fig. 1). On the treated specimen, the *p*-layer doping contrast was markedly enhanced by up to 50% for high acceptor concentrations (N_a_) > 10^19^ cm^−3^ but reduced by up to threefold otherwise, and resulting in more than double the sensitivity to N_a_ changes (specificity). The surface band-bending is strong in heavily doped regions due to an increased surface state density, thereby imparting (attenuating) kinetic energy for SEs escaping the *p*-type (*n*-type) surface^13^, and hence boosting doping contrast. For moderate to light doping, the internal surface fields are weak, and extend deeper below the surface^13^; but the surface band-bending effect on SEs (within the escape depth), which although differentiates the doping level, is not expected to be as significant^13,26^ as that of the patch fields (see later). Consequently, doping contrast is reduced since the surface Fermi level pinning results in decreased surface potentials or patch fields, which are in turn essentially independent of the doping^13,29^. Moreover, this doping contrast reduction may further imply quantitative characteristics of the pre-existing doping-dependent surface oxide film^39^ especially in the doping range ≤ 10^18^ cm^−3^.

Comparing freshly-cleaved specimens *a* and *b*, doping contrast disparities for a given N_a_ are due to the influence of doping geometry-dependent patch fields with or without the thin silicon-germanium epitaxial marker layer^26^. Within experimental error, there is practically no difference in the doping contrast from treated surfaces of heavily doped regions (*eg*. 6 × 10^18^ cm^−3^), and this is attributed to strong surface band-bending which suppresses the patch field effects^27^, as will be illustrated in the ensuing SE spectral imaging results.

### Energy-filtered SE imaging and *in situ* spectromicroscopy

#### Freshly-cleaved silicon specimens

**Figure 2:**
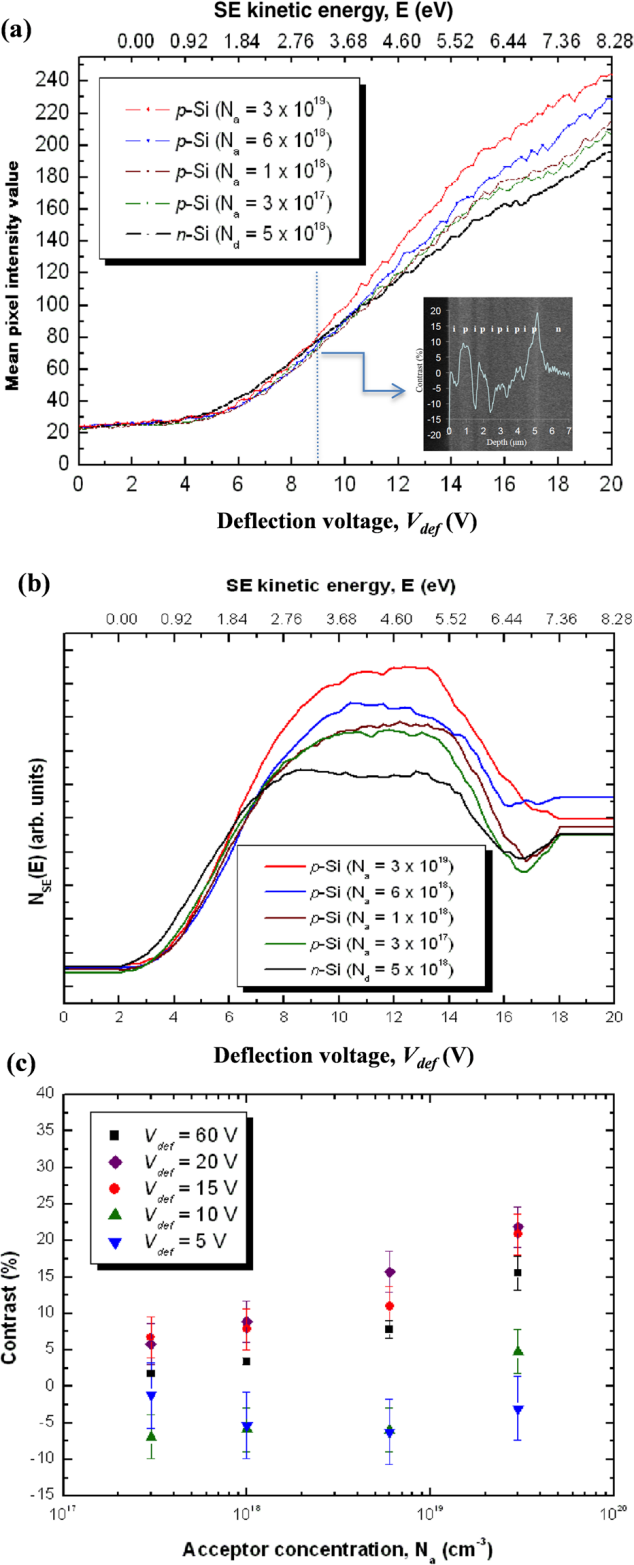
Doping contrast analysis by energy-filtering on the freshly-cleaved silicon specimen. (**a**) Experimental SE intensity curves from the respective *p*- and *n*-regions (*c.f*. specimen *b* of Fig. 1); inset shows an energy-filtered cross-sectional SE image of the sample at *V*_*def*_ = 9 V, including a doping contrast profile overlay. (**b**) Differential SE spectra. (**c**) Column-averaged doping contrast values as a function of N_a_ for *V*_*def*_ at 5, 10, 15 and 20 V (filtered; *c.f*. Fig. 2(a)), and 60 V (unfiltered).

Figure 2(a) shows experimental SE intensity curves (energy integrals of the spectra) from the respective doped regions in specimen *b* (*c.f*. Fig. 1), including an inset at *V*_*def*_ = 9 V demonstrating contrast inversion for layer III (~10^18^ cm^−3^) but nearly zero contrast for layers II (~6 × 10^18^ cm^−3^) and IV (~3 × 10^17^ cm^−3^). The extraction potential was 250 V and working distance was ~3 mm, so that the high extraction field maximises the number of SEs by increasing the angular acceptance range into the lens bore for the more energetic particles^29^. However, a larger extraction voltage and/or smaller working distance increases the detector collection solid angle which in turn reduces doping contrast; patch field effects can be more fully accounted for and quantification accuracy is enhanced^26,29^, though at the price of significant doping contrast because SEs emitted at higher angles contribute less to doping information^13^. The SE intensity curve from the *n*-substrate intersects with that from all the *p*-layers at the respective *V*_*def*_ (see Fig. 2(a)), indicating contrast inversion onset at ~8.62 ± 0.04 and ~11.02 ± 0.04 V for the highest and lowest N_a_ respectively. Whilst patch fields, and hence the doping contrast (unfiltered), progressively increase with N_a_, energy-filtering reveals a general decrease in the contrast inversion onset energy.

Despite the energy upshift of the *p*-layer integral curve relative to that of the *n*-substrate, the low energy emission does not relate to the energy shift-based model system invoked by Kazemian *et al*.^21,22^ for measuring surface potentials. It is understood that the region doped to a higher N_a_ is at a lower potential, thus the ionisation energy is smaller. Therefore secondary emissions from a region of higher N_a_ should in principle have a higher average kinetic energy. If energy-filtering of the SEs were employed allowing through only the least energetic electron flux, the image intensity should stem preponderantly from the region of low N_a_. Hence in energy-filtered images, lower N_a_ regions should accordingly appear brighter. Nevertheless, a converse result was demonstrated incontrovertibly by our experiments (see Fig. 2).

The SE intensity is largely incommensurate at *V*_*def*_ < 5 V, but an inverse correlation between the energy displacement and N_a_ is clear in the upper energy portion (*e.g*. > 6.21 eV) of the yield curve (Fig. 2(a)). Qualitative agreement is found for the latter relationship with measurements in ref.^21^ exhibiting energy upshifts of the SE intensity curve as N_a_ reduces (or surface potential increases). However, this is supposably peripheral to their main results because it fundamentally undermines the ability to calibrate the SE intensity effect of changing *V*_*def*_ on silicon samples using the *negatively correlated* copper wire bias-mediated spectral shifts. Crucially, the dissimilar secondary emission properties from bulk silicon and copper and their associated surface dipoles might have been overlooked, leading somewhat to a major theoretical aberration. Positive covariance of the spectral shift with N_a_ is exhibited in the differential spectra within a higher, albeit limited, energy band above the peak energies (Fig. 2(b)), but this arises from broader spectral emission with increasing acceptor doping. Furthermore, the presence of significant patch fields, surface oxide/contamination or micro-roughness results in poorly-defined energy peaks between 2.76 ± 0.01 and 5.52 ± 0.01 eV through their influence on the emission and trajectories of the secondaries.

Two- to three-fold reduction in the unfiltered doping contrast is indicated in Fig. 2(c) (*c.f.* Fig. 1) due to a higher angular collection efficiency of the SEs, but in the same figure we demonstrate that energy-selective imaging, *e.g*. at 15 V ≤ *V*_*def*_ ≤ 20 V, can enhance the sensitivity by at least ~40%, and specificity by up to ~66%, for the doping range studied. Though, the classical logarithmic doping dependence of contrast approximation is not satisfied when energy-filtering at *V*_*def*_ ≤ 10 V. Whilst the doping contrast displayed non-monotonicity using the slowest secondaries, it can be seen that contrast inversion occurred on every *p*-layer at *V*_*def*_ = 5 V, and on *p*-layers with N_a_ ≤ 6 × 10^18^ cm^−3^ at *V*_*def*_ = 10 V (see Fig. 2(a) and (c)).

#### Surface-treated silicon specimens

**Figure 3:**
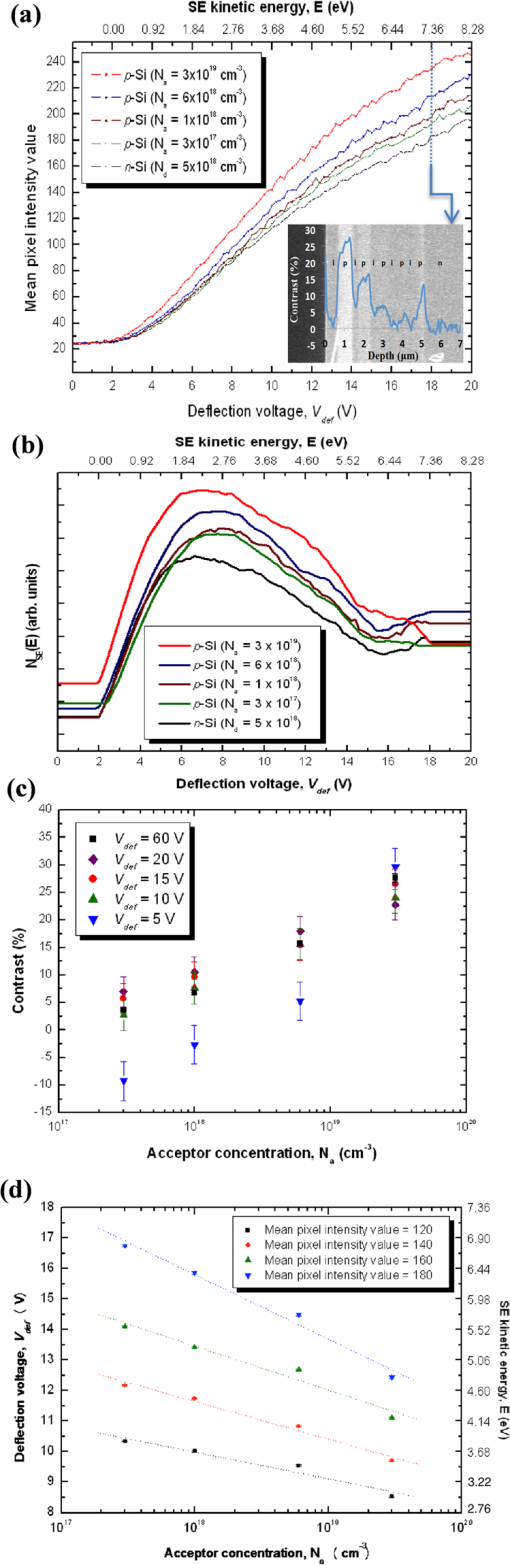
Doping contrast analysis by energy-filtering on the NH_4_F-treated silicon specimen. (**a**) Experimental SE intensity curves from the respective *p*- and *n*-regions (*c.f*. specimen *b* of Fig. 1); inset shows an energy-filtered cross-sectional SE image of the sample at *V*_*def*_ = 18 V, including a doping contrast profile overlay. (**b**) Differential SE spectra. (**c**) Column-averaged doping contrast values as a function of N_a_ for *V*_*def*_ at 5, 10, 15 and 20 V (filtered; *c.f*. Fig. 3(a)), and 60 V (unfiltered). (**d**) SE kinetic energy-calibrated *V*_*def*_ for 8 V ≤ *V*_*def*_ ≤ 18 V versus N_a_ at specific intensity levels (*c.f*. Fig. 3 (a)).

Unlike that from the freshly-cleaved specimen, basically no contrast inversion of the *p*-layer occurred for N_a_ ≥ 10^18^ cm^−3^ after surface-treatment (see Fig. 3(a)). The SE intensity curves from the *p-*layers I to III converge with that from the *n*-substrate (*i.e*. zero contrast) at *V*_*def*_ = 1.80 ± 0.02 to 7.24 ± 0.02 V for the highest to the lowest N_a_ respectively. Hence, the low energy emission characteristics signify the suppression of patch fields due to Fermi level pinning by a high surface state density^13,27^. Strong surface band-bending and a significantly reduced surface junction potential is commensurate with dopant concentration, so that the increased (decreased) secondary emission rate at the *p*-type (*n*-type) region is evident over the entire *V*_*def*_ range wherein the *p*-layer yield curve is seen to translate towards lower energies with respect to that of the *n*-substrate as N_a_ increases. Nevertheless, the *p*-layer IV curve intersects with that of the *n*-substrate at *V*_*def*_ values between 7.60 ± 0.02 and 8.20 ± 0.02 V, with contrast inversion occurring below which till 3.46 ± 0.02 V, thereby suggesting that the surface band-bending for N_a_ ≤ 3 × 10^17^ cm^−3^ is insufficient to quench out patch field effects on the slowest secondaries^13,27^. Accordingly, the *p*-layer IV differential spectrum upshifted in energy with respect to that of the *n*-substrate, contrary to that of the other *p*-layers (see Fig. 3(b)). The energy peaks have also become more pronounced and have concomitantly reduced to between 1.93 ± 0.01 and 2.51 ± 0.01 eV.

For quantification purposes, there is also a wider spectral range for which the SE intensity depends monotonically on N_a_, notably when energy-filtering at *V*_*def*_ > 3.56 ± 0.02 V (Fig. 3(a) and (c)), though at *V*_*def*_ ≥ 10 V there is no considerable impact on doping contrast, even for low N_a_ (see Fig. 3(c)). The intensities/contrast appear similar to that as-cleaved at *V*_*def*_ = 20 V (*c.f*. Fig. 2(c)), thereby suggesting a correlation with surface band-bending within the relevant escape depths rather than any patch fields (now diminished) or any extraneous effects due to a previously reactive or microscopically rough surface. At low *V*_*def*_ (*e.g*. 5 V), the results depict two putative cases: (i) for the lowest N_a_ (*e.g*. 3 × 10^17^ cm^−3^) regions, the slowest SEs, which are sensitive to any surface potential difference, stem mainly from the *n-*region (or higher potential surface), hence yielding contrast inversion and; (ii) for sufficiently high N_a_ (*e.g*. 3 × 10^19^ cm^−3^), the strong surface band-bending boosts emission of even the least energetic SEs, thus inhibiting contrast inversion^13^ (*c.f*. Fig. 3(a) and (c)).

The correspondence between *V*_*def*_ and N_a_ at different mean pixel intensities (Fig. 3(d)) show that to obtain a higher SE count, the kinetic energy limit has to be increased, and vice versa. The data are described quite well by the linear regressions (dotted lines), which demonstrate that to obtain a given number of secondaries, the maximum kinetic energy requirement increases monotonically as N_a_ reduces (or *V*_*def*_  → *A* V as N_a_ → 1 cm^−3^) according to the following relation:

1 $${N}_{a}={10}^{-(\frac{{V}_{def}-A}{B})}$$

Moreover, the greater the pixel intensity value, the larger the *V*_*def*_ change (or *B*) is required to equalise the SE count between different N_a_; this result, which characterises the integrated SE spectral shift, can be derived from a wider *V*_*def*_ range after surface-treatment, and in this sense, means an increased spectral resolution. Therefore the empirical *V*_*def*_ dependence of the SE intensity may confer predictive capability to evaluate the doping provided that it is calibrated by a quantitative model such as that based on the Monte Carlo method taking into account patch fields, surface band-bending, surface boundary scattering and the detector solid collection angle as addressed above, and elaborated in ref.^13^.

## Discussion

Angle- and energy-resolved doping contrast measurements and *in situ* SE spectromicroscopy studies have for the first time been performed, analysed and compared from a wide doping range, with and without surface-treatment of the silicon specimens. Doping contrast under a controlled low and high angular/energy collection efficiency of the detector has illustrated the classic roles of patch fields and surface band-bending. The slowest secondaries, which are most sensitive to any patch fields, originate mainly from the *n*-region (having higher ionisation energy) for freshly-cleaved specimens. Hence when energy-filtering, contrast inversion occurs due to convolution of the angular acceptance and patch field effects^13^. The extent of negative correlation with N_a_ of the low energy range for which the *n*-region SE yield eclipses that of the *p*-region indicates the competing effects of surface band-bending and patch fields (Fig. 2(a)); surface band-bending effects, which are strong at high doping concentrations, prevail above the threshold *V*_*def*_ and vice versa. After etching in NH_4_F, surface Fermi level pinning was induced by a high density of surface states, which suppresses patch fields and largely isolates surface band-bending effects on doping contrast^27^. The increased (decreased) number of secondaries emitted from the *p*-(*n-)*region, even for the slowest particles, explains why contrast inversion generally does not occur (see Fig. 3(a)).

On account of the non-linear surface band-bending dependence of the SE energy and angular distributions, the linear analytical model applied by Kazemian *et al*.^21,22^ is likely to be inadequate to accurately determine the surface potentials of *p*-*n* junctions since the doping-dependent field and scattering effects are governed by the three-dimensional (sub-)surface potential disposition. Moreover, their theory^21,22^ essentially avers that doping contrast is zero when patch fields are suppressed or when no surface potential variation exists across surface Fermi level pinned homo-/hetero-junctions^18,22^. However, we expect that even though a homogenous net charge density or in-plane uniformity of surface states characterises the surface plane, doping sensitive surface dipoles or surface band-bending will yet play a considerable role in doping contrast generation^13,27^. The surface band-bending effects are reflected in the spectral peak broadening with increasing acceptor concentration (*c.f*. Figs 2(b) and 3(b)) as the surface depletion width becomes comparable with the SE escape depth^13,26,29^. Hence for quantitative dopant profiling, instead of quantifying only the singular spectral shift in a linear regime^21,22^, we recommend mapping out the one-to-one correspondence between the intensity/contrast and dopant concentration at the respective *V*_*def*_, and analysing the spectromicroscopy results through a detailed comparison with numerical simulations in refs^13,27^ as we have done in the present study.

Additionally, we have demonstrated that energy-filtering conspicuously enhances the dopant profiling sensitivity and specificity for freshly-cleaved specimens, albeit choosing from within a limited spectral band for accurate quantification (Fig. 2(c)). By energy-filtering, signal components selected from within a depth below the surface evade deleterious effects once ineluctable from oxides, Si-SiO_2_ interfaces, beam-induced contamination or surface micro-roughness; but too low *V*_*def*_ renders quantification impracticable as the least energetic SEs succumb to the relatively strong geometry-dependent patch fields. After NH_4_F-treatment, the doping contrast reduces when below a critical doping concentration (see Fig. 1), which we attribute to total internal reflection and Fresnel losses owing to an amplified index contrast^13,29^ after removal of the surface oxide layer; and increases otherwise when the former is superseded by surface band-bending effects at higher doping concentrations (>10^18^ cm^−3^). Moreover, the logarithmic N_a_ dependence of the doping contrast strikingly manifests within a much wider energy range (Fig. 3(c)). Passing through only the slowest secondaries (*e.g*. ≤1.38 eV) can further enhance sensitivity to N_a_ changes by exploiting the competing relationship between patch fields and surface band-bending. For high N_a_, remnant patch fields are insignificant due to proportionately strong surface band-bending, and the converse is true. Information complementary to the aforementioned is unravelled in the spectromicroscopy measurements that unambiguously indicate the potential of surface-treatment to facilitate quantitative dopant profiling over a broader spectral range after dissolution of any oxides/contamination, reduction in rugosity, passivation of the surface, and suppression of the patch fields. Consequently, the spectral peaks are more pronounced, and a concomitant redshift can be seen in the differential spectra (Fig. 3(b)), thereby reflecting a systematic dependence on the surface electron affinity modified by surface band-bending due to hydrogen atom surface termination and Fermi level pinning. Above a critical *V*_*def*_, energy-filtering has practically little impact on doping contrast (Fig. 3(c)) as surface band-bending effects outcompete that of patch fields, and thus may not be crucial for quantification after appropriate surface hydrogen passivation and termination.

In this study, the foregoing results underscore the prospects of optimising surface-treatment to enable targeted and proportionate doping contrast from clean and passivated surfaces that is purely differentiated by doping-dependent surface band-bending, and the cardinal importance of calibrating the edge terminations so that accurate dopant profiling is possible by means of a robust, quantitative model accounting for surface states and surface band-bending.

## Materials and Methods

### Doping contrast characterisation from abrupt homojunctions

The multilayered specimens comprise a series of boron-doped (*p*-type) layers having thicknesses and dopant levels ranging from ~0.4 to 0.8 μm and ~3 × 10^17^ to 7 × 10^19^ cm^−3^ respectively, on a monocrystalline *n*-type silicon substrate antimony-doped to 5 × 10^18^ cm^−3^, with or without a 100 ± 0.01 nm thick epitaxial marker layer of silicon-germanium (with 5 at.% Ge). The capping layer and the spacer layers between the *p*-layers were nominally undoped. These specimens were diced from 5-inch silicon wafers that were chemical vapour deposition (CVD)-grown along the [001] direction with dopant incorporation at a growth rate of ~0.15 μm/min., under an operating temperature of ~1123 K and atmospheric pressure.

Spectral imaging and SE doping contrast measurements were performed on silicon {110} cross-sections that were freshly-cleaved in ambient air, and directly after surface-treatment by aqueous NH_4_F solution. Great care was taken to ensure that the cleaved surface was optically flat and mirror-like, and clean and of high quality, without evident striations in the regions of interest. During surface-treatment, the samples were dipped into freshly-prepared 40% NH_4_F (ARISTAR™) for ~1 min., using PTFE tweezers, before they were rinsed thoroughly in deionised (DI) water for ~3–4 min. to reduce ammonium salt deposits on the surface. The highly hydrophobic treated surface implies at least partial hydrogen-termination. The specimen was inserted into the SEM vacuum chamber with intervening exposure to air of no more than ~3 min. The base pressure was ~3 × 10^–6^ mbar using an oil-free turbo-pump system.

Mechanical alignments were carefully made so that the primary electron beam was incident normally on the specimen cross-section, and the doped homojunctions were orthogonal to the raster scan direction. A 30 μm-diameter objective aperture was used, with an optimised set of beam parameters for doping contrast: a 1 kV focussed electron probe having a current of ~32 pA and a probe diameter of ~16 nm. SE imaging was performed in the ultra-high resolution (UHR) mode, and to ensure high contrast and signal-to-noise ratio, all the images (712 × 484 pixels) were digitally acquired at a magnification of 8000 × and a scan frequency of ~0.1 frame s^−1^, and stored as 8-bit datasets. Too high a magnification and too low a scan frequency may impair doping contrast through excess electron-hole pair generation and surface charging, and/or contamination. The data were processed using a Java plug-in written for ImageJ^40^. Line profiles across regions of interest were row-averaged over at least 100 pixels perpendicular to the scan direction to obtain the doping contrast profiles. The doping contrast value *C* was determined by normalising the SE intensity from the region of interest to that from the doped substrate through the following formalism:

2 $$C=\{\begin{array}{c}\frac{\Delta I}{{\dot{I}}_{d}}=\frac{{I}_{d}-{\overline{I}}_{sub}}{{I}_{d}-{I}_{0}};\,{I}_{d}\ge {\overline{I}}_{sub}\\ \frac{\Delta I}{{\dot{I}}_{sub}}=\frac{{I}_{d}-{\overline{I}}_{sub}}{{\overline{I}}_{sub}-{I}_{0}};\,{I}_{d} < {\overline{I}}_{sub}\end{array}$$

*I*_*d*_ is the pixel intensity from the layer of interest, ${\bar{I}}_{sub}$ is the mean pixel intensity from the uniformly doped substrate, and *I*_0_ is the spurious background intensity obtained by blanking out the primary electron beam. The absolute contrast value lies within −1 ≤ *C* ≤ 1, and is independent of the contrast and brightness settings on the microscope control console.

The SEM was a Schottky field emission gun (sFEG) FEI XL30^TM^ equipped with an SE detection system that combines a through-the-lens detector (TLD) with an energy filter. Under standard UHR imaging conditions, the deflection voltage (*V*_*def*_) is set at a maximum of 60 V, and image formation and doping contrast are derived from SEs of virtually all energies (up to 50 eV), which are trapped on-axis by the strong objective lens magnetic fields, and spiral up through the lens bore at a rate proportionate to the extraction voltage. To perform low-pass energy-filtering, *V*_*def*_ is reduced, the magnitude of which dictates the highest kinetic energies of the SEs that are allowed to pass through to the scintillator to be detected and contribute to the image; further elaboration on energy-filtering with the TLD can be found in refs^21,29^.

### SE energy-filtering and *in situ* spectromicroscopy

By steadily varying *V*_*def*_ in discrete steps at regular temporal intervals, SE spectral measurements can be directly performed via energy-filtering for the evaluation of surface potentials and surface states^27^. High spectral resolution was enabled by a bespoke DC power supply that can configure on the deflector electrode, potential changes as small as ±30 mV over a dynamic range from 0 to 20 V^21,29^. *V*_*def*_ control and real-time data acquisition were automated through the National Instruments (NI) DAQ platform, which utilises data I/O and a timing controller with LabVIEW 7^TM^ software to synchronously acquire, process and analyse data on a separate personal computer. The pixel intensity value at each deflector bias step characterises the energy integral of the SEs up to the corresponding maximum kinetic energy limit; differentiating the SE intensity with respect to *V*_*def*_ results in its spectral distribution. For quantitative analysis, the SE intensity measurements were processed by box averaging at least 40 × 40 pixels within targeted scanned field areas at a magnification of 6500× and a TV scan frequency of 1 frame _8×_ s^−1^ (frame_8×_ refers to 8 averaged frames), and recorded in real-time against *V*_*def*_ which was stepped through a voltage/energy range of interest. Beam-induced extraneous effects were kept minimal with a judicious choice of a dwell time <400 ns as complete data acquisition was performed rapidly (within 50 s) under the scan settings used. Essentially the same yield curves were obtained when forward or reverse sweeping *V*_*def*_ in 0.2 V steps, thereby confirming that a delicate balance between granularity and throughput is struck of the discrete temporal sequence to curtail contamination/charging effects on the measurements over the full *V*_*def*_ range for the specimens studied; and doping contrast is not a dynamical, but statical, function of *V*_*def*_.

To calibrate the energy response of the TLD, we leveraged our Monte Carlo method^13^ to compute its geometrical acceptance by ray tracing the SEs from a point source of a Lambertian angular distribution, through immersion lens magnetic and electrostatic fields in the electron optical column and the specimen chamber. These latter fields are described by a generic, geometric finite-element model of the TLD at the specified UHR settings, including extraction voltage, working distance, *etc*., for spectral imaging^27^. An ideal detector is assumed, having a step function discriminator response, 100% intrinsic efficiency of the scintillator/photomultiplier, and ergodicity of pixel response. Disregarding the tertiary electron (SE3) signals originating from the pole pieces or objects other than the sample, a linear detector response function, DRF = $\frac{\Delta {V}_{def}}{\Delta {E}_{SE}}$ is found in the low-energy portion (at least <12 eV) with which the maximum kinetic energy (*E*_*SE*_) limits are mapped onto *V*_*def*_. The actual sensitivity of the energy-resolved detector is further limited by the threshold energy for SE detection, and the origin of kinetic energy is estimated to be centred on *V*_*def*_  ≈ 2 V, which depends not only on the geometrical acceptance, but the type and thickness of the protective passivation layer on the scintillator.

## References

[1] Liu P, Lee J, Huan Y, Su D (2009). Application of secondary electron potential contrast on junction leakage isolation. Appl. Phys. Lett..

[2] Liu P, Lee J (2011). Inspection of the current-mirror mismatch by secondary electron potential contrast with *in situ* nanoprobe biasing. IEEE Elec. Dev. Lett..

[3] Rosenkranz R (2011). Failure localization with active and passive voltage contrast in FIB and SEM. J. Mat. Sci.: Mat. Elec..

[4] Lee J, Liu P (2012). Surface potential mapping of p^+^/n-well junction by secondary electron potential contrast with *in situ* nano-probe biasing. Microelec. Eng..

[5] Xu L (2013). Secondary electron microscopy dopant contrast image (SEMDCI) for laser doping. IEEE J. Photovolt..

[6] Moldovan G (2007). Low-voltage cross-sectional EBIC for characterisation of GaN-based light emitting devices. Ultramicrosc..

[7] Oatley C, Everhart TE (1957). The examination of p-n junctions with the scanning electron microscope. J. Elec. and Contr..

[8] Elliott S, Broom R, Humphreys C (2002). Dopant profiling with the scanning electron microscope—A study of Si. J. Appl. Phys..

[9] Tsurumi D, Hamada K, Kawasaki Y (2012). Energy-filtered secondary-electron imaging for nanoscale dopant mapping by applying a reverse bias voltage. Jap. J. Appl. Phys..

[10] Jatzkowski J, Simon-Najasek M, Altmann F (2012). Novel techniques for dopant contrast analysis on real IC structures. Microelec. Rel..

[11] Zhu Y, Inada H, Nakamura K, Wall J (2009). Imaging single atoms using secondary electrons with an aberration-corrected electron microscope. Nat. Mat..

[12] Shibata N (2017). Electric field imaging of single atoms. Nat. Comm..

[13] Chee AKW, Bosch EGT, Broom RF, Humphreys CJ (2011). A quantitative model for doping contrast in the scanning electron microscope using calculated potential distributions and Monte Carlo simulations. J. Appl. Phys..

[14] Castell MR (1995). Topographical, compositional, and dopant contrast from cleavage surfaces of GaAs AlxGa1xAs superlattices. J. Phys. Conf. Ser..

[15] Perovic DD (1995). Field-emission SEM imaging of compositional and doping layer semiconductor superlattices. Ultramicrosc..

[16] Wager, J. & Kuhn, K. Device physics modeling of surfaces and interfaces from an induced gap state perspective. *Crit. Rev. Sol. Stat. Mat. Sci*., 1–43 (2016).

[17] Howie A (1995). Recent developments in secondary electron imaging. J. Microsc..

[18] Sealy CP, Castell MR, Wilshaw PR (2000). Mechanism for secondary electron dopant contrast in the SEM. J. Elec. Microsc..

[19] Ciappa M, Ilgünsatiroglu E, Illarionov A (2012). Monte Carlo simulation of emission site, angular and energy distributions of secondary electrons in silicon at low beam energies. Microelec. Rel..

[20] Buzzo M, Ciappa M, Fichtner W (2006). Imaging and dopant profiling of silicon carbide devices by secondary electron dopant contrast. IEEE Trans. on Dev. and Mat. Rel..

[21] Kazemian P, Mentink S, Rodenburg C, Humphreys C (2006). High resolution quantitative two-dimensional dopant mapping using energy-filtered secondary electron imaging. J. Appl. Phys..

[22] Kazemian P, Mentink S, Rodenburg C, Humphreys C (2007). Quantitative secondary electron energy filtering in a scanning electron microscope and its applications. Ultramicrosc..

[23] Tsurumi D, Hamada K, Kawasaki Y (2010). Energy-filtered imaging in a scanning electron microscope for dopant contrast in InP. J. Elect. Microsc..

[24] Tsurumi D, Hamada K, Kawasaki Y (2012). Highly reproducible secondary electron imaging under electron irradiation using high-pass energy filtering in low-voltage scanning electron microscopy. Microsc. and Microanal..

[25] Chee AKW, Boden S (2016). Dopant profiling based on scanning electron and helium ion microscopy. Ultramicrosc..

[26] Chee AKW (2016). Quantitative dopant profiling by energy filtering in the scanning electron microscope. IEEE Trans. Dev. and Mat. Rel..

[27] Chee AKW (2016). Fermi level pinning characterisation on ammonium fluoride-treated surfaces of silicon by energy-filtered doping contrast in the scanning electron microscope. Sci. Rep..

[28] El-Gomati, M., Wells, T., Mullerova, I., Frank, L. & Jayakody, H. Why is it That Differently Doped Regions in Semiconductors are Visible in Low Voltage SEM? *IEEE Trans. Electron Dev.* **51**, 288–291 (2004).

[29] Chee, A. K. W. Novel investigations of contrast in the scanning electron microscope towards a new generation of dopant profiling techniques engineered for semiconductor (opto)electronic device technology. PhD thesis (University of Cambridge, Cambridge, United Kingdom, 2009).

[30] Rivillon S, Chabal YJ, Amy F, Kahn A (2005). Hydrogen passivation of germanium (100) surface using wet chemical preparation. Appl. Phys. Lett..

[31] Koenraad, P. & Flatté, M. Single dopants in semiconductors. *Nat. Mat.* **10**, 91–100 (2011). 10.1038/nmat2940 21258352

[32] Higashi, G., Becker, R., Chabal, Y. & Becker, A. Comparison of Si(111) surfaces prepared using aqueous solutions of NH4F versus HF. *Appl. Phys. Lett.* **58**, 1656–1658 (1991).

[33] Allongue, P., Kieling, V. & Gerischer, H. Etching mechanism and atomic structure of H-Si(111) surfaces prepared in NH4F. *Electrochim. Act.* **40**, 1353–1360 (1995).

[34] Angermann, H., Rappich, J., Sieber, I., Hübener, K. & Hauschild, J. Smoothing and passivation of special Si(111) substrates: studied by SPV, PL, AFM and SEM measurements.* Anal. Bioanal. Chem.* **390**, 1463–1470 (2008). 10.1007/s00216-007-1738-5 18066540

[35] Chabal, Y., Higashi, G., Raghavachari, K. & Burrows, V. Infrared spectroscopy of Si(111) and Si(100) surfaces after HF treatment: Hydrogen termination and surface morphology. *J. Vac. Sci. Tech. A: Vac., Surf., Films.* **7**, 2104–2109 (1989).

[36] Morita, M., Ohmi, T., Hasegawa, E., Kawakami, M. & Suma, K. Control factor of native oxide growth on silicon in air or in ultrapure water. *Appl. Phys. Lett.* **55**, 562–564 (1989).

[37] Houston, M. & Maboudian, R. Stability of ammonium fluoride‐treated Si(100). *Journal of Applied Physics* **78**, 3801–3808 (1995).

[38] Gonda, S., Tanaka, M., Kurosawa, T. & Kojima, I. Sub-nanometer scale measurements of silicon oxide thickness by spectroscopic ellipsometry. *Japanese Journal of Applied Physics* **37**, L1418–L1420 (1998).

[39] Duraud, J. P., Le Moël, A., Le Gressus, C., Pantel, R. & Chornik, B. Contrast of a p-n junction in ultra high vacuum. *Scanning Elect. Microsc.* **1**, 49–54 (1984).

[40] Schneider, C. A., Rasband, W. S. & Eliceiri, K. W. NIH Image to ImageJ: 25 years of image analysis. *Nat. Methods.* **9**, 671–675 (2012). 10.1038/nmeth.2089 PMC5554542 22930834

