# Author Correction: Enhancing doping contrast and optimising quantification in the scanning electron microscope by surface treatment and Fermi level pinning

**DOI:** 10.1038/s41598-020-76566-5

**Published:** 2020-11-17

**Authors:** Augustus K. W. Chee

**Affiliations:** 1 Centre for Advanced Photonics and Electronics, Electrical Engineering Division, Department of Engineering, University of Cambridge, 9 JJ Thomson Avenue, Cambridge, CB3 0FA United Kingdom; 2 Department of Materials Science and Metallurgy, University of Cambridge, Pembroke Street, Cambridge, CB2 3QZ United Kingdom

Correction to: *Scientific Reports* https://doi.org/10.1038/s41598-018-22909-2, published online 27 March 2018

In the original version of this Article, an additional affiliation for Augustus K. W. Chee was omitted. In addition, a present address was inadvertently included and has now been removed. The correction affiliations are:

Centre for Advanced Photonics and Electronics, Electrical Engineering Division, Department of Engineering, University of Cambridge, 9 JJ Thomson Avenue, Cambridge CB3 0FA, United Kingdom.

Department of Materials Science and Metallurgy, University of Cambridge, Pembroke Street, Cambridge CB2 3QZ, United Kingdom.

The version of this Article previously published also contained a low resolution Figure 1.

In addition, the original version of this Article contained typographical errors.

In the Introduction section,

“Further, by acquiring an image series through an energy window of interest,* in situ* SE spectromicroscopy mandates an additional dimension permitting evaluation of the kinetic energy constituents of the composite signal^22,27^.”

now reads:

“Further, by acquiring an image series through an energy window of interest,* in situ* SE spectromicroscopy mandates an additional dimension permitting evaluation of the surface electronic band structure via kinetic energy (*E*_SE_) constituents of the composite signal^22,27^.”

In the Materials and Methods section, under subheading ‘Doping contrast characterisation from abrupt homojunctions’,

“*I *_*d*_ is the column-averaged SE intensity from the layer of interest, ${\overline{I}}_{sub}$ is the mean SE intensity from the uniformly doped substrate, and *I*_0_ is the spurious background intensity obtained by blanking out the primary electron beam.”

now reads:

“*I *_*d*_ is the pixel intensity from the layer of interest, ${\overline{I}}_{sub}$ is the mean pixel intensity from the uniformly doped substrate, and *I*_0_ is the spurious background intensity obtained by blanking out the primary electron beam.”

In the same section, under subheading ‘SE energy-filtering and* in situ* spectromicroscopy’,

“The actual sensitivity of the energy-resolved detector is further limited by the threshold energy for SE detection, and the origin of kinetic energy is estimated to be centred on *V *_*def*_ ≈ 2 V, which depends not only on the geometrical acceptance, but the type and thickness of the protective passivation layer on the detector.”

now reads:

“The actual sensitivity of the energy-resolved detector is further limited by the threshold energy for SE detection, and the origin of kinetic energy is estimated to be centred on *V *_*def*_ ≈ 2 V, which depends not only on the geometrical acceptance, but the type and thickness of the protective passivation layer on the scintillator.”

In the legend of Figure 1,

“(Bottom) Row-averaged doping contrast values from the *p*-layers of interest as a function of acceptor concentration, before and after surface-treatment.”

now reads:

“(Bottom) Column-averaged doping contrast values from the *p*-layers of interest as a function of acceptor concentration, before and after surface-treatment.”

In the legend of Figure 2,

“(**c**) Doping contrast as a function of N_a_ for *V *_*def*_ at 5, 10, 15 and 20 V (filtered; *c.f*. Fig. 2(a)), and 60 V (unfiltered).”

now reads:

“(**c**) Column-averaged doping contrast values as a function of N_a_ for *V *_*def*_ at 5, 10, 15 and 20 V (filtered; *c.f*. Fig. 2(a)), and 60 V (unfiltered).”

In the legend of Figure 3,

“(**c**) Doping contrast as a function of N_a_ for *V *_*def*_ at 5, 10, 15 and 20 V (filtered; *c.f*. Fig. 3(a)), and 60 V (unfiltered).”

now reads:

“(**c**) Column-averaged doping contrast values as a function of N_a_ for *V *_*def*_ at 5, 10, 15 and 20 V (filtered; *c.f*. Fig. 3(a)), and 60 V (unfiltered).”

In the Acknowledgements,

“The author thanks Professor Sir Colin Humphreys (University of Cambridge), Professor Peter Wilshaw (University of Oxford), Dr John Ellis (X-FAB Silicon Foundries) and Dr Heike Angermann (Helmholtz-Zentrum Berlin) for useful discussions, and FEI company for initiating the Image Contrast and Detection (ICD) European academic-industrial consortium programme that forms the subject of this manuscript.”

now reads:

“The author thanks Professor Sir Colin Humphreys (University of Cambridge), Professor Peter Wilshaw (University of Oxford), Dr John Ellis (X-FAB Silicon Foundries) and Dr Heike Angermann (Helmholtz-Zentrum Berlin) for useful discussions, and Dr Gerard Van Veen and Dr Seyno Sluyterman (FEI company) for initiating the Image Contrast and Detection (ICD) European academic-industrial consortium programme that forms the subject of this manuscript.”

These errors have now been corrected in the HTML and PDF versions of the Article.

